# Optical *in vivo* imaging detection of preclinical models of gut tumors through the expression of integrin αVβ3

**DOI:** 10.18632/oncotarget.25826

**Published:** 2018-07-31

**Authors:** Giulia Marelli, Roberta Avigni, Paola Allavena, Cecilia Garlanda, Alberto Mantovani, Andrea Doni, Marco Erreni

**Affiliations:** ^1^ IRCCS Humanitas Clinical and Research Center, Rozzano, Milan, Italy; ^2^ Humanitas University, Rozzano, Milan, Italy; ^3^ The William Harvey Research Institute, Queen Mary University of London, London, UK; ^4^ Current address: Center for Molecular Oncology, Bart Cancer Institute, Queen Mary University of London, London, UK

**Keywords:** optical imaging, FMT, intestine tumor, integrin αVβ3, colorectal cancer

## Abstract

Optical imaging and Fluorescent Molecular Tomography (FMT) are becoming increasingly important for the study of different preclinical models of cancer, providing a non-invasive method for the evaluation of tumor progression in a relatively simple and fast way. Intestinal tumors, in particular colorectal cancer (CRC), represent a major cause of cancer-related death in Western countries: despite the presence of a number of preclinical models of intestinal carcinogenesis, there is a paucity of information about the possibility to detect intestinal tumors using fluorescent probes and optical *in vivo* imaging. Herein, we identify the detection of integrin αvβ3 by FMT and optical imaging as an effective approach to assess the occurrence and progression of intestinal carcinogenesis in genetic and chemically-induced mouse models. For this purpose, a commercially available probe (IntegriSense), recognizing integrin αvβ3, was injected in APC^+/min^ mice bearing small intestinal adenomas or CRC: FMT analysis allowed a specific tumor detection, further confirmed by subsequent *ex vivo* imaging or conventional histology. In addition, IntegriSense detection by FMT allowed the longitudinal monitoring of tumor growth. Taken together, our data indicate the possibility to use integrin αvβ3 for the visualization of intestinal tumors in preclinical models.

## INTRODUCTION

Colorectal cancer (CRC) is a frequent human neoplasia and still one of the major leading causes of cancer-related death, especially in Western Countries [1]. Both transplanted orthotopic and spontaneous mouse models of colorectal cancer are available, representing a fundamental tool to understand the biological mechanisms of tumor progression and to evaluate the efficacy of potential therapeutic approaches. However, to assess disease state without sacrificing the animals, only indirect parameters are commonly used, such as animal weight loss, food and water uptake, stool consistency or the presence of occult blood in the feces. On the other hand, invasive procedures such as conventional optical endoscopy can be operated, but with the risk of perforating the colon and bleeding, thus impacting on the quality of the analysis [2]. In addition, a 6–27% miss rate recognition of small polyps have been estimated when samples are analysed by white light colonoscopy [3]. Different molecular *in vivo* imaging methods are now available, allowing the detection and the non-invasive characterization of tumor development in living animals, reducing the number of mice required per experiments and increasing the statistical power of the analysis. In preclinical oncological studies, in addition to conventional imaging techniques such as positron emission tomography (PET), magnetic resonance imaging (MRI), computed tomography (CT) and ultrasounds, optical imaging procedures can be used for the visualization of biological processes in small animal models expressing specific reporters (luciferase or fluorescent proteins) or injected with specific fluorescent probes [4–7]. However, a major drawback of conventional two-dimensional optical imaging is that the acquired signal is attenuated as much as the light source is deeper embedded in tissue. In this context, Fluorescent Molecular Tomography (FMT), a relatively new optical imaging technology, significantly improves the quality and the spatial resolution of optical imaging analysis, being able to localize and, more importantly, quantify fluorescent probes three-dimensionally in deep tissues at high sensitivity [8, 9].

The characterization of processes involved in neoplastic progression and the description of tumor features are of primary importance to determine the correct cancer staging and to identify the best prognostic, predictive or therapeutic approach for oncologic patients. In this scenario, the expression of integrins, has been demonstrated to influence the prognosis in various cancer types [10]. Integrins are small transmembrane heterodimeric glycoproteins involved in cell adhesion, cell-matrix interaction and cell signalling pathways, whose expression has been correlated with tumor cell migration and metastatic processes [11]. Among members of this family, integrin αvβ3 is one of the most extensively studied and has been associated with the early phase of angiogenesis in a variety of tumors, including colorectal cancer [12, 13]. In addition, studies demonstrated the efficacy of targeting integrin αvβ3 for the imaging identification and therapeutic treatment of several tumors, such as breast cancer, lung cancer and glioblastomas [14, 15].

In this study, we provide evidence that imaging of integrin αvβ3 can be successfully used for the visualization and detection of intestinal tumors in preclinical models. The methodology described here may represent an evaluable tool to analyse the pathogenesis of intestinal tumors and therapeutic drug development.

## RESULTS

### *In vivo* detection by αvβ3 integrin expression of tumor formation in the small intestine

To test the efficacy of targeting integrin αvβ3 to visualize spontaneous genetic model of small intestine tumors, we took advantage of the APC^+/min^ (APC, Adenomatous Polyposis Coli) mouse model. APC^+/min^ mice are characterized by a single point mutation in the murine homolog of the *APC* gene. These mice develop multiple adenomas in the small intestine, representing a very powerful and commonly used model to study intestinal carcinogenesis [16]. In addition, APC^+/min^ mice were previously crossed with mice lacking the expression of the chemokine receptor CX_3_CR1, known to have a role in regulating intestinal inflammation and whose absence induces intestinal carcinogenesis [17–20], in order to generate mice either competent (APC^+/min^-CX_3_CR1^+/–^) or not (APC^+/min^-CX_3_CR1^–/–^) for CX_3_CR1 expression and carrying concomitantly the mutation in *APC* gene. The double-mutant mice allowed us to compared not only tumor-bearing mice with healthy subjects, but also mice with a different tumor-load, due to the CX_3_CR1-dependent phenotype.

**Figure 1:**
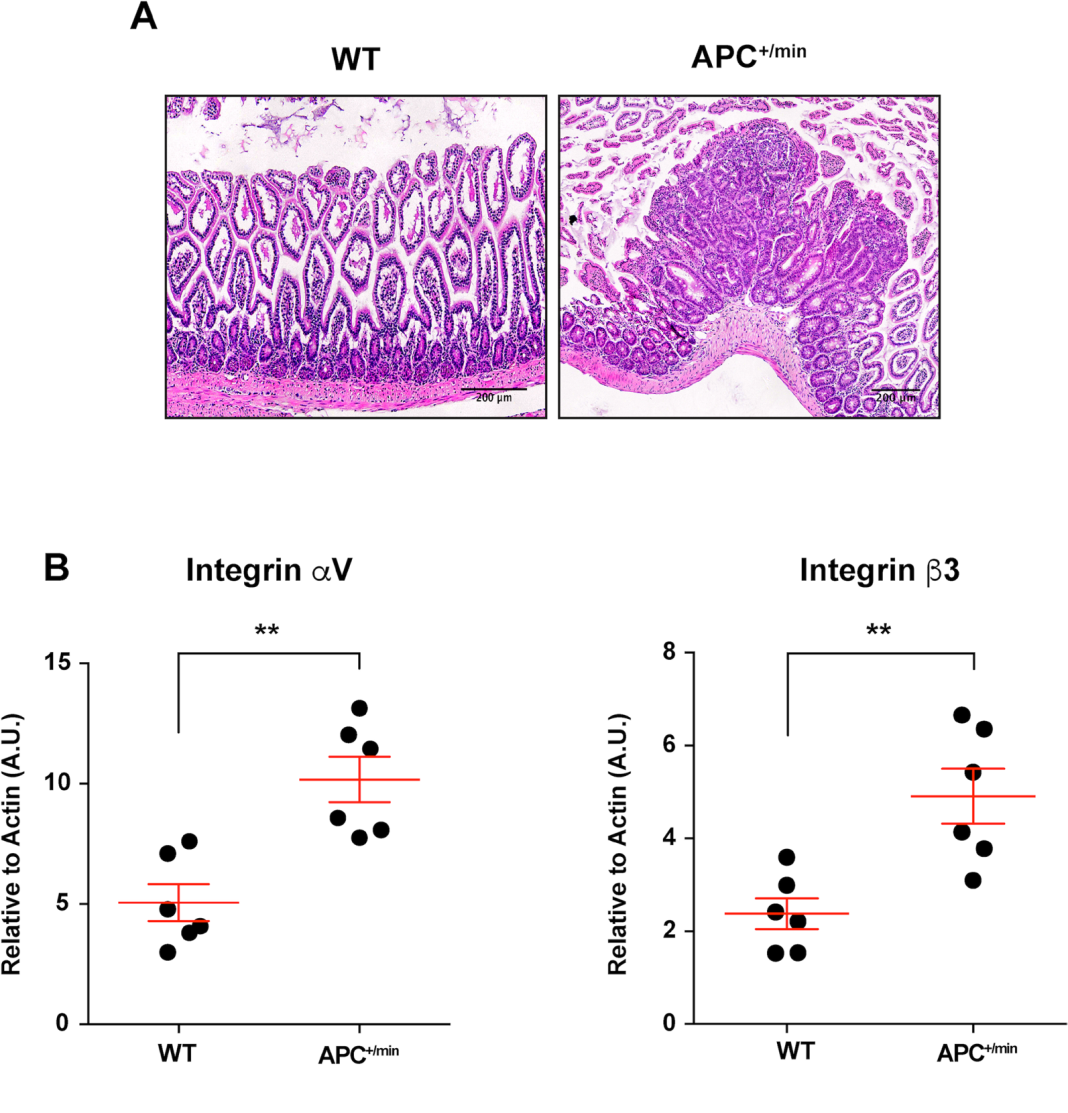
Integrin αVβ3 expression in small intestine of 18 weeks old APC^+/min^ and WT mice (**A**) Histological analysis of small intestine tumor occurrence in 18 weeks old APC^+/min^ mice and age-related WT mice. Original magnification 10×. (**B**) Evaluation of mRNA expression of αV and β3 integrin subunit by qPCR in healthy (WT) and tumor tissue (APC^+/min^). Data are presented as Mean ± SEM. Student’s *t* Test, ^**^p < 0.01.

The occurrence of small intestine tumors was analysed by histology in 18 weeks-old APC^+/min^ mice and age-related WT mice (Figure 1A). Subsequently, we investigated the expression of integrin αvβ3 in small intestine tumors. We excised tumor lesions or corresponding healthy mucosa from APC^+/min^ and WT mice, respectively: mRNA analysis by q-PCR indicated that the levels of both αv and β3 subunits were significantly upregulated in small intestine tumors in comparison to corresponding healthy mucosa (Figure 1B).

**Figure 2:**
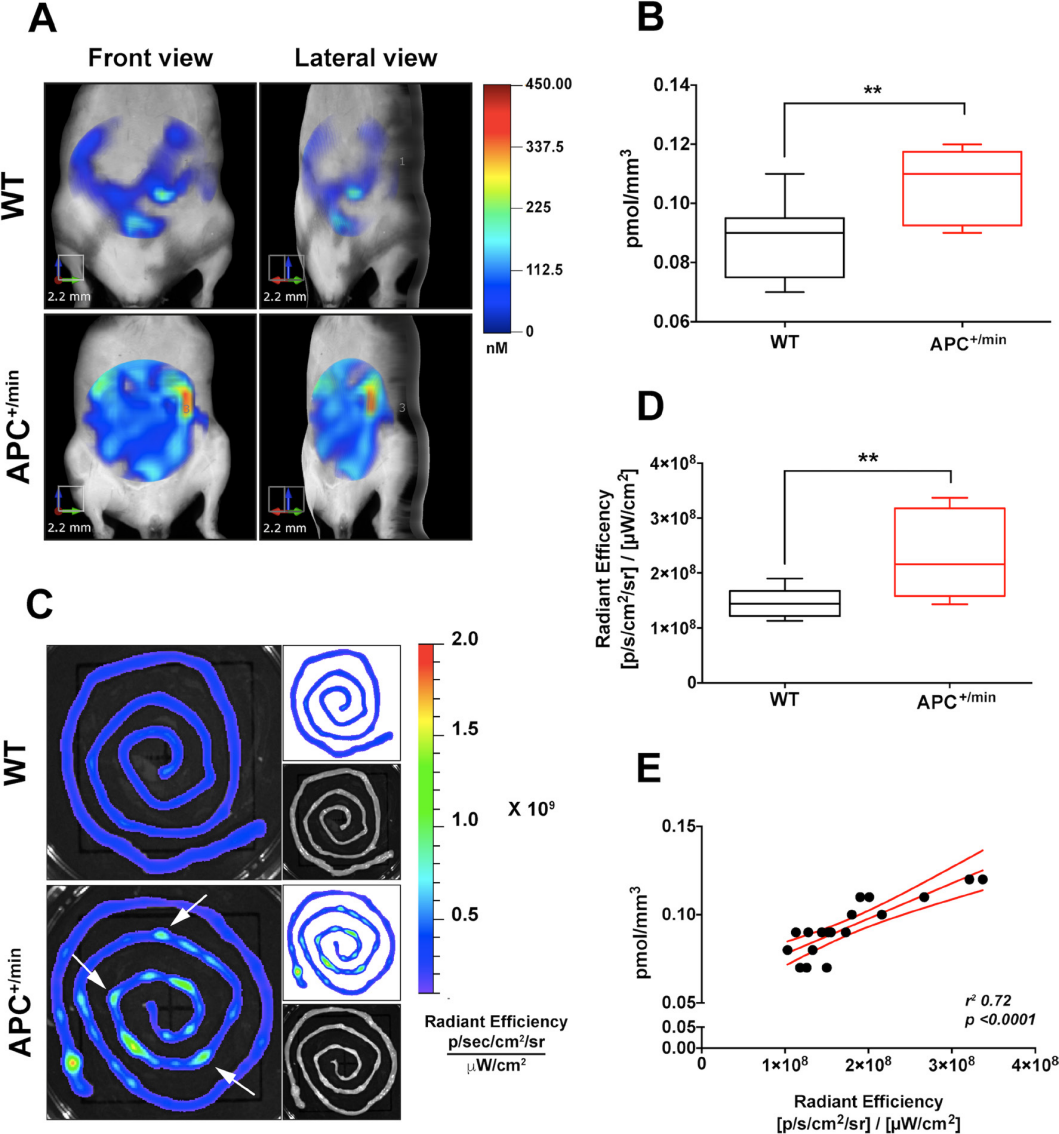
*In vivo* and *ex vivo* imaging detection of small intestine tumor by IntegriSense750 probe (**A**) *In vivo* Fluorescent Molecular Tomography (FMT) analysis of IntegriSense750 accumulation in the small intestine of 18 weeks old APC^+/min^ and WT mice. Region of interest (ROI) was tri-dimensionally defined to outline the small intestine. The colorbar shows the concentration (in nM) of the IntegriSense750 probe. (**B**) Quantification of FMT analysis of IntegriSense750 probe accumulation in the small intestine of 18 weeks old APC^+/min^ and WT mice. The total amount of probe within the ROI (in pmoles) was calculated as indicated in the M&M. The amount of pmoles of the probe was normalized based on the volume (mm^3^) of the ROI, after adjustment using the statistically derived threshold value. Data are presented as min and max whiskers plot. Student’s *t* Test, ^**^p < 0.01. (**C**) *Ex vivo* analysis of the small intestine of APC^+/min^ and WT mice previously acquired by FMT. The colorbar shows the Radiant Efficiency. White arrows indicate the accumulation of IntegriSense750 probe in the neoplastic lesions. (**D**) Quantification of the *ex vivo* analysis of IntegriSense750 probe accumulation in the small intestine of APC^+/min^ and WT mice. Data are presented as min and max whiskers plot. Student’s *t* Test, ^**^p < 0.01 (**E**) Correlation between *in vivo* FMT quantification (pmol/mm^3^) and *ex vivo* quantification (Radiant Efficiency) of IntegriSense750 accumulation in the small intestine of both APC^+/min^ and WT mice. Data are presented as Mean and Error (95% CI). Linear regression was calculated (*r*^*2*^ *0.72, p < 0.0001*).

To evaluate whether the expression of the integrin αvβ3 could be used to detect small intestine tumor formation *in vivo*, 24 h before the imaging, 18 weeks-old APC^+/min^ mice and age-related WT mice were shaved and injected IV with 0.08 nmol/g of body weight of IntegriSense750 (Perkin Elmer), a commercial available probe specifically recognizing integrin αvβ3 [21] and emitting a near infrared (NIR) fluorescent signal at 750 nm wavelength. Animals have been fed with the AIN76A, alfalfa-free, rodent diet (Mucedola srl) for 2 weeks, in order to minimize the autofluorescence originating from the animals’ intestinal contents. Mice were positioned in a dedicated imaging cassette and IntegriSense750 signal acquired with the fluorescent molecular tomography (FMT) imaging system FMT2000 (Perkin Elmer). A 3D ellipsoidal region of interest (ROI) was outlined to select, as much as possible, the small intestine. Subsequently, we quantified probe concentration (as pmol/mm^3^) within the ROI: independently from the CX_3_CR1 status, IntegriSense accumulated in the small intestine and this accumulation strongly increased in small intestine of APC^+/min^ mice compared to WT mice (Figure 2A and 2B).

To further confirm the specificity of the *in vivo* signal acquisition, both APC^+/min^ and WT mice were sacrificed and small intestine collected for *ex vivo* imaging analysis, performed with the IVIS Lumina III optical imaging system (Perkin Elmer). *Ex vivo* analysis confirmed the increased accumulation of IntegriSense750 probe in the small intestine of tumor-bearing APC^+/min^ mice compared to WT mice (Figure 2C and 2D): of note, several spots of probe accumulation can be identified in the small intestine of APC^+/min^ mice (Figure 2C, white arrows), likely corresponding to the localization of the developed multiple small adenomas. To further verify the accuracy and the correct positioning of the *in vivo* ROIs, we correlated the *in vivo* quantification of the IntegriSense750 probe and *ex vivo* acquired signal, finding a significant correlation between *in vivo* and *ex vivo* results (*r*^*2*^ *0.72, p < 0.0001*), thus confirming the specificity of the detection (Figure 2E). In conclusion, APC^+/min^ tumor-bearing mice show a higher accumulation of integriSense750 probe in comparison to WT mice, suggesting that more fluorescent signal corresponds to an increased tumor load in APC^+/min^ mice.

**Figure 3:**
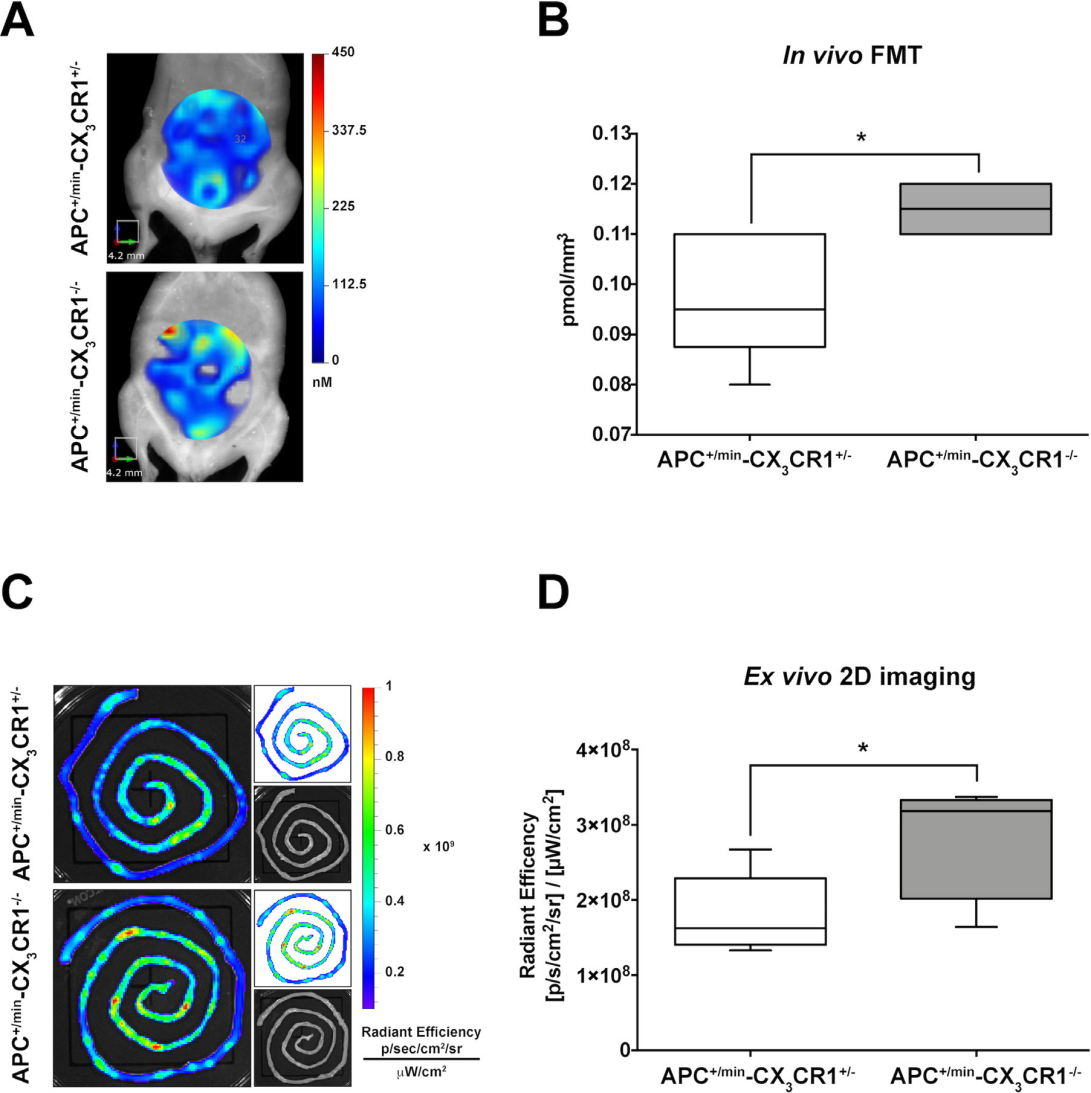
*In vivo* and *ex vivo* imaging analysis of IntegriSense750 accumulation in the small intestine of APC^+/min^-CX_3_CR1^+/-^ and APC^+/min^-CX_3_CR1^-/-^ mice (**A**) *In vivo* Fluorescent Molecular Tomography (FMT) analysis of IntegriSense750 accumulation in the small intestine of 18 weeks old APC^+/min^-CX_3_CR1^+/-^ and APC^+/min^-CX_3_CR1^-/-^ mice. Region of interest (ROI) was tri-dimensionally defined to outline the small intestine. The colorbar shows the concentration (in nM) of the IntegriSense750 probe. (**B**) FMT quantification of IntegriSense750 probe in the small intestine of APC^+/min^-CX_3_CR1^+/-^ and APC^+/min^-CX_3_CR1^-/-^ mice. The total amount of probe within the ROI (in pmoles) was calculated relative to the internal standard generated with a known concentration of the appropriate NIR dye, following manufacturer’s instruction. The amount of pmoles of the probe was normalized based on the volume (mm^3^) of the ROI, after adjustment using the statistically derived threshold value. Data are presented as min and max whiskers plot. Student’s *t* Test, ^*^p < 0.05 (**C**) *Ex vivo* analysis of the small intestine of APC^+/min^-CX_3_CR1^+/-^ and APC^+/min^-CX_3_CR1^-/-^ mice previously acquired by FMT. The colorbar shows the Radiant Efficiency. (**D**) *Ex vivo* quantification of the IntegriSense750 probe in the small intestine of APC^+/min^-CX_3_CR1^+/-^ and APC^+/min^-CX_3_CR1^-/-^ mice. Data are presented as min and max whiskers plot. Student’s *t* Test, ^*^p < 0.05.

Finally, given the role of CX_3_CR1 in regulating intestinal inflammation, we compared the formation of small intestine adenomas in APC^+/min^-CX_3_CR1^+/-^ and APC^+/min^-CX_3_CR1^-/-^. When the CX_3_CR1 status was considered, we found that APC^+/min^-CX_3_CR1^-/-^ mice showed a slightly but significant increased accumulation of IntegriSense750 probe compared to APC^+/min^-CX_3_CR1^+/-^ mice both in *in vivo* FMT and *ex vivo* imaging analysis (Figure 3A, 3B and Figure 3C, 3D, respectively), confirming our (and other) previous results of increased tumor occurrence in CX_3_CR1-deficient mice [20, 22]. This result indicates that IntegriSense750 is able not only to distinguish tumor tissue from healthy mucosa, but also to discriminate between different tumor load in small intestine adenomas-bearing mice.

### *In vivo* detection of tumor formation in the colon by αvβ3 integrin expression

**Figure 4:**
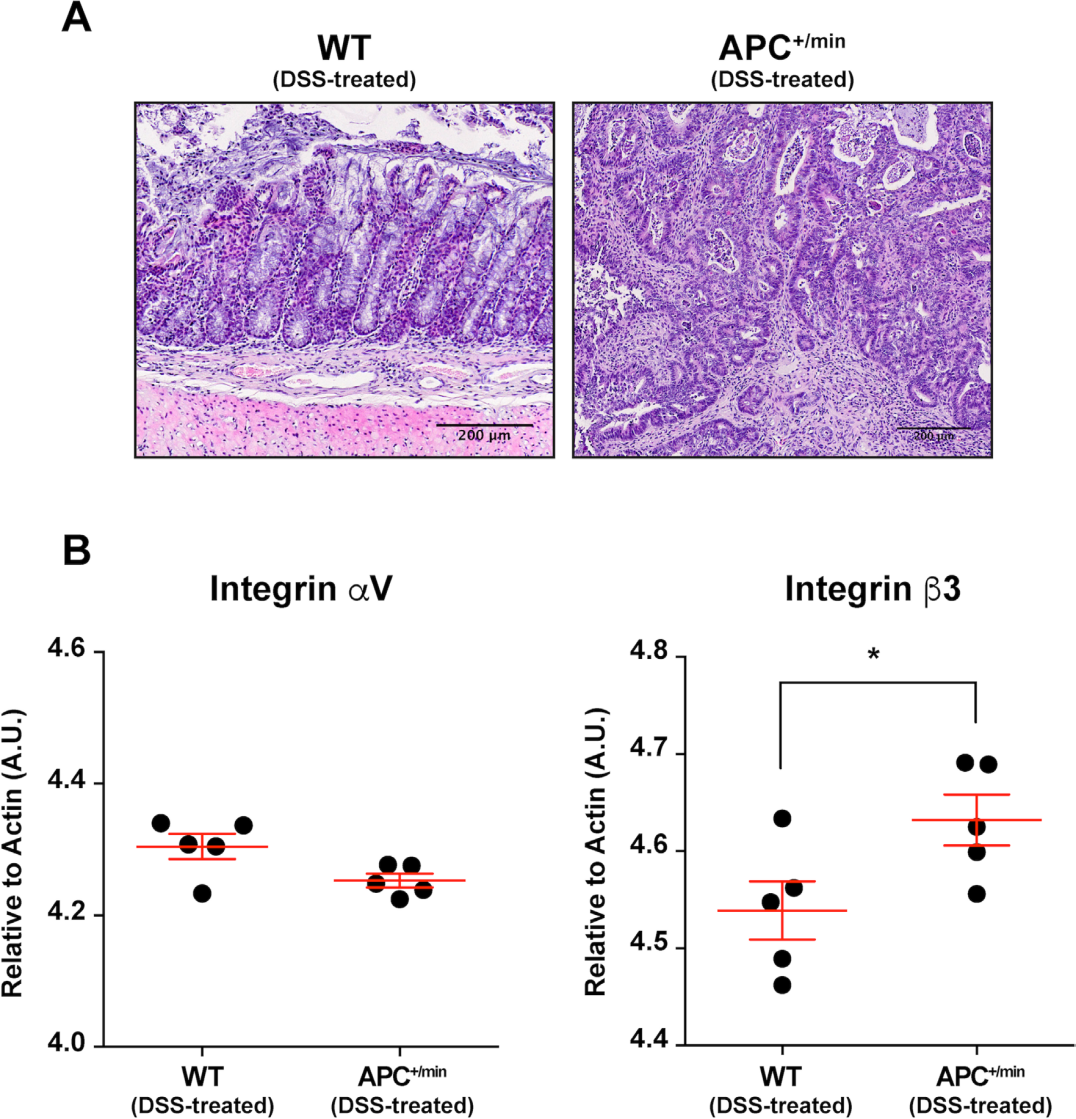
Integrin αVβ3 expression in the colon of DSS-treated APC^+/min^ and DSS-treated WT mice (**A**) Histological analysis of colorectal tumor occurrence in APC^+/min^ mice and age-related WT mice after dextran sulfate sodium (DSS) administration (as described in the “Results” section). Original magnification 10× (**B**) Evaluation of mRNA expression of αV and β3 integrin subunit by qPCR in healthy (WT) and tumor (APC^+/min^) tissue. Data are presented as Mean ± SEM. Student’s *t* Test, ^*^p < 0.05.

We subsequently evaluated the feasibility of targeting integrin αvβ3 in a chemically-induced mouse model of colon cancer. APC^+/min^ mice spontaneously develop multiple adenomas in the small intestine but few in the colon. To induce the formation of colorectal tumors, APC^+/min^ and WT mice were administered with dextran sulfate sodium (DSS) in drinking water: DSS is a widely used irritant of the colonic mucosa, able to cause intestinal inflammation (in a B- and T-cell independent manner), likely through the damaging of epithelial monolayer lining the large intestine and the consequent dissemination of pro-inflammatory luminal content into the tissue [23]. We administered 3% DSS to 4 weeks-old APC^+/min^ and WT mice for 7 days, followed by 4 weeks of fresh water [20, 24]. The occurrence of tumors was assessed by histology, comparing colon from APC^+/min^ and WT DSS-treated mice (Figure 4A). Subsequently, we evaluated the mRNA expression of integrin αvβ3. We excised tumor lesions or corresponding healthy mucosa from the colon of DSS-treated APC^+/min^ and DSS-treated WT mice, respectively: mRNA analysis by qPCR indicated a significant increase in the expression of the β3 subunit in tumor lesions of DSS-treated APC^+/min^ mice (Figure 4B).

**Figure 5:**
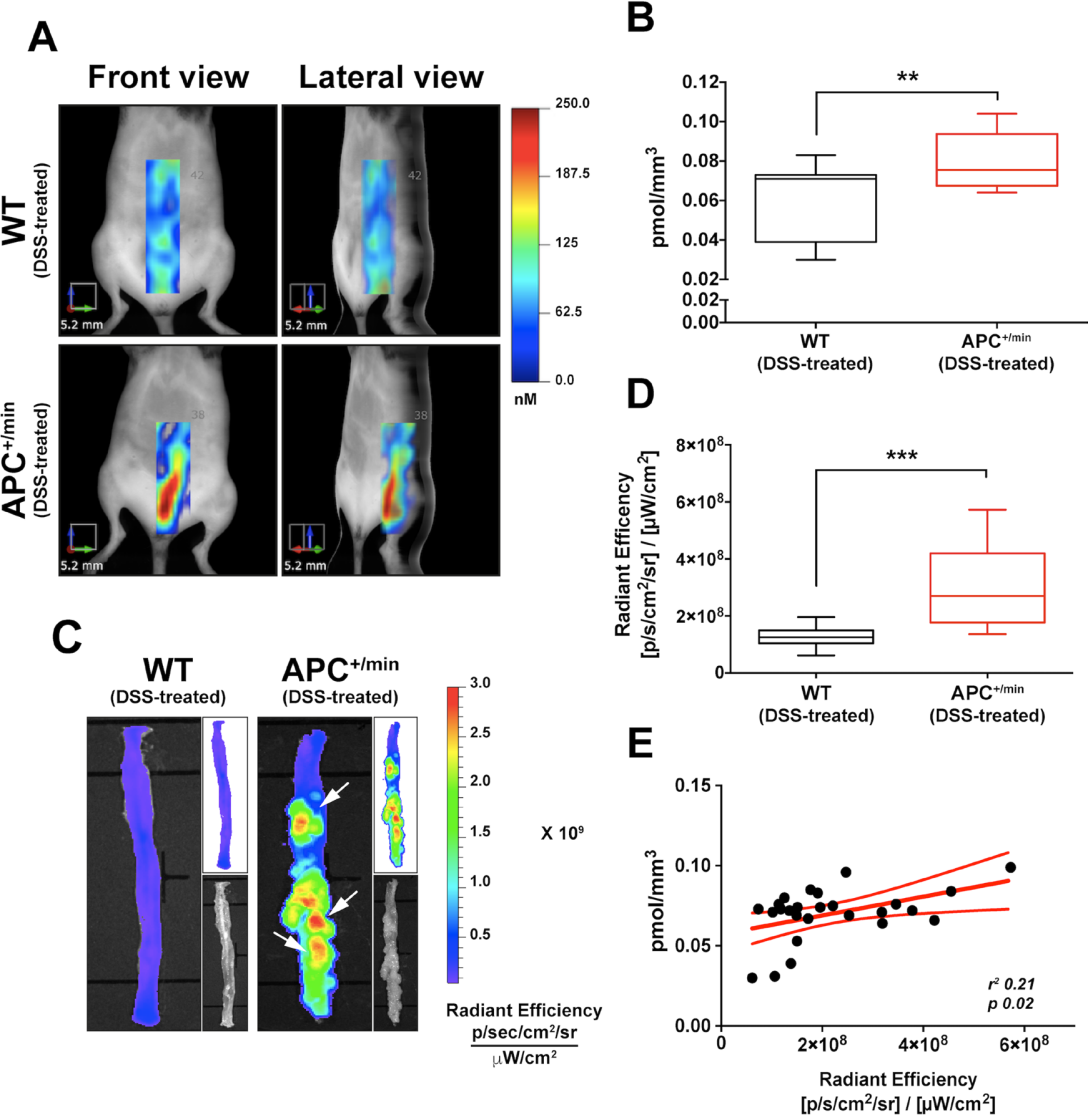
*In vivo* and *ex vivo* imaging detection of colorectal tumor by IntegriSense750 probe (**A**) *In vivo* Fluorescent Molecular Tomography (FMT) analysis of IntegriSense750 accumulation in the colon of the DSS-treated APC^+/min^ and DSS-treated WT mice. Region of interest (ROI) was tri-dimensionally defined to outline the colon. The colorbar shows the concentration (in nM) of the IntegriSense750 probe. (**B**) Quantification of FMT analysis of IntegriSense750 probe accumulation in the colon of DSS-treated APC^+/min^ and DSS-treated WT mice. The total amount of probe within the ROI (in pmoles) was calculated as indicated in M&M. The amount of pmoles of the probe was normalized based on the volume (mm^3^) of the ROI, after adjustment using the statistically derived threshold value. Data are presented as min and max whiskers plot. Student’s *t* Test, ^**^p < 0.01 (**C**) *Ex vivo* analysis of the colon of DSS-treated APC^+/min^ and DSS-treated WT mice previously acquired by FMT. The colorbar shows the Radiant Efficiency. White arrows indicate the accumulation of IntegriSense750 probe in the tumor lesions. (**D**) Quantification of the *ex vivo* analysis of IntegriSense750 probe accumulation in the colon of DSS-treated APC^+/min^ and DSS-treated WT mice. Data are presented as min and max whiskers plot. Student’s *t* Test, ^***^p < 0.001 (**E**) Correlation between *in vivo* FMT quantification (pmol/mm^3^) and *ex vivo* quantification (Radiant Efficiency) of IntegriSense750 accumulation in the colon of DSS-treated APC^+/min^ and DSS-treated WT mice. Data are presented as Mean and Error (95% CI). Linear regression was calculated (*r*^*2*^ *0.21, p = 0.02*).

We then evaluated *in vivo* the expression of integrin αvβ3. 24 h before the acquisition, mice were shaved and injected IV with 0.08 nmol/g of body weight of IntegrinSense750. Also in this case, animals have been fed for 2 weeks with AIN76A, alfalfa-free, rodent diet. Mice were positioned in the dedicated imaging cassette and IntegriSense750 signal acquired with FMT2000 system. A cylindrical 3D region of interest (ROI) was outlined in order to select, as much as possible, the colon. Independently from the CX_3_CR1 status, probe quantification revealed higher accumulation of the IntegriSense750 in the colon of DSS-treated APC^+/min^ mice compared to DSS-treated WT mice (Figure 5A and 5B).

Subsequently, both APC^+/min^ and WT DSS-treated mice were sacrificed and colon collected for *ex vivo* imaging analysis by IVIS system, clearly confirming the increased concentration of IntegriSense750 probe in the colon of APC^+/min^ mice compared to WT mice (Figure 5C and 5D): in particular, IntegriSense750 accumulation specifically corresponds to the visible colonic neoplastic lesions of APC^+/min^ DSS-treated mice (Figure 5C). Also in this case, even if the *r*^*2*^ is poor, a significant linear correlation was found between the *in vivo* FMT concentration of the IntegriSense750 probe and *ex vivo* IVIS acquired signal, confirming the specificity of the detection (*r*^*2*^ *0.21, p < 0.02,* Figure 5E).

Finally, we compared the formation of colorectal tumors in APC^+/min^-CX_3_CR1^+/-^ and APC^+/min^-CX_3_CR1^-/-^ DSS-treated mice: when the CX_3_CR1 status was considered, we did not find any difference in the IntegriSense750 accumulation between APC^+/min^-CX_3_CR1^+/-^ and APC^+/min^-CX_3_CR1^-/-^ mice (data not shown).

### Correlation between *in vivo* and *ex vivo* αvβ3 integrin detection and histological analysis

**Figure 6:**
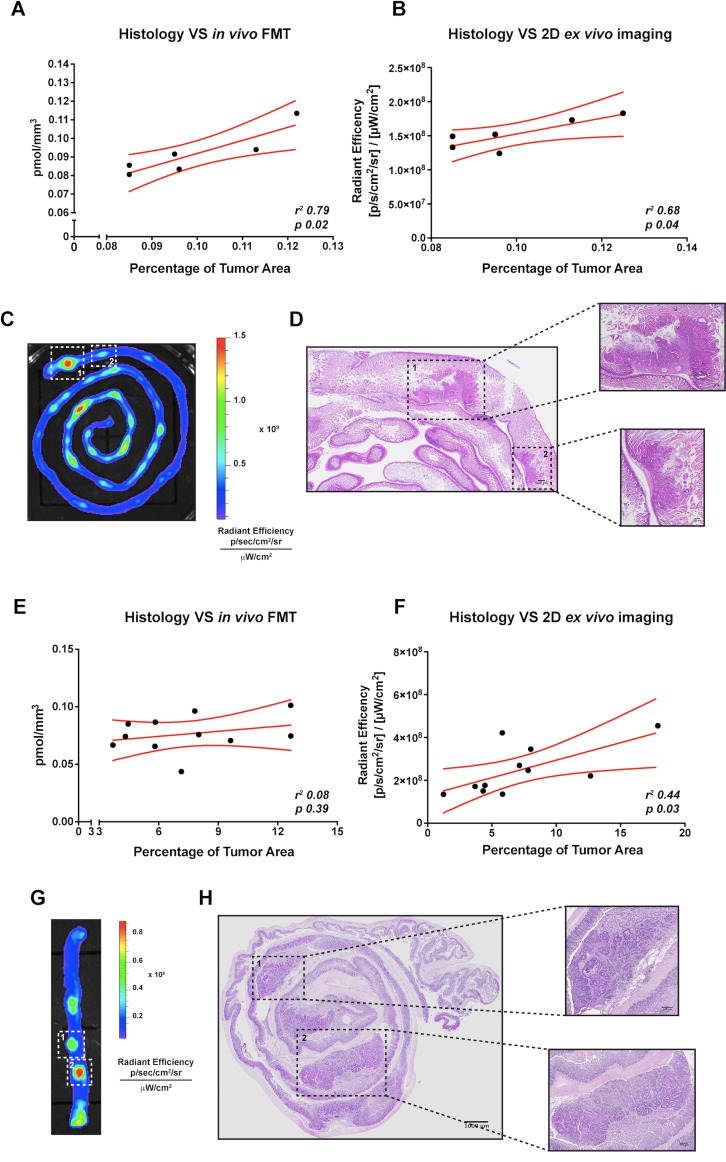
Correlation between *in vivo* or *ex vivo* imaging analysis and conventional histology of small intestine and colorectal tumor-bearing APC^+/min^ mice (**A**) Correlation between FMT IntegriSense750 probe quantification (pmol/mm^3^) and the percentage of tumor area calculated by histological analysis (as indicated in M&M) in small intestine tumor-bearing APC^+/min^ mice. Data are presented as Mean and Error (95% CI). Linear regression was calculated (*r*^*2*^ *0.79, p = 0.02*). (**B**) Correlation between *ex vivo* imaging analysis of IntegriSense750 probe accumulation and the percentage of tumor area calculated by histological analysis (as indicated in M&M) in small intestine tumor-bearing APC^+/min^ mice. Data are presented as Mean and Error (95% CI). Linear regression was calculated (*r*^*2*^ *0.68, p = 0.04*). (**C**, **D**) Representative image of small intestine tumors identified by *ex vivo* imaging (C) and conventional histology (D). White-dotted line squares in panel C and black-dotted line squares in panel D indicate the same neoplastic lesions. Original magnification 10× (**E**) Correlation between FMT IntegriSense750 probe quantification (pmol/mm^3^) and the percentage of tumor area calculated by histological analysis (as indicated in M&M) in colon tumor-bearing DSS-treated APC^+/min^ mice. Data are presented as Mean and Error (95% CI). Linear regression was calculated (*r*^*2*^ *0.08, p = 0.39*). (**F**) Correlation between *ex vivo* imaging analysis of IntegriSense750 probe accumulation and the percentage of tumor area calculated by histological analysis (as indicated in M&M) in colon tumor-bearing DSS-treated APC^+/min^ mice. Data are presented as Mean and Error (95% CI). Linear regression was calculated (*r*^*2*^ *0.44, p = 0.03*). (**G**, **H**) Representative image of colorectal tumors identified by *ex vivo* imaging (G) and conventional histology (H). White-dotted line squares in panel G and black-dotted line squares in panel H indicate the same neoplastic lesions. Original magnification 10×.

Histology still represents the leading technique for the evaluation of tumor occurrence and dimension. We therefore compared the results obtained from the *in vivo* and *ex vivo* imaging acquisition with the histological analysis. Small intestine tumor and colon cancer from mice previously analysed for the expression of IntegriSense750 probe were collected and stained with hematoxylin and eosin. Sections were scanned with VS120-S5 Virtual Slide System (Olympus). Subsequently, we calculated the percentage of tumor area as described in “Material and Methods”. We then correlated the results obtained from the *in vivo* and *ex vivo* imaging analysis with the percentage of tumor area. Interestingly, we found a significant correlation between the small intestine tumor area and the quantification of IntegriSense750 acquired both *in vivo* with FMT (*r*^*2*^ *0.79, p 0.02*, Figure 6A) and *ex vivo* with the IVIS system (*r*^*2*^ *0.68, p 0.04,* Figure 6B). In particular, some spots of IntegriSense750 accumulation distinguished with the *ex vivo* analysis corresponded to small adenomas identified by conventional histological analysis (Figure 6C, white-dotted line squares, and Figure 6D).

The same correlation was performed on colon tumors. In this case, a significant correlation was found only between histological analysis and the *ex vivo* acquisition with IVIS system (*r*^*2*^ *0.44, p 0.03,* Figure 6F), even if a tendency was observed also for the FMT analysis (*r*^*2*^ *0.08, p 0.39,* Figure 6E). As for the small intestine tumors, the spots of IntegriSense750 accumulation detected by *ex vivo* imaging analysis matched with colonic tumors recognizable by conventional histological analysis (Figure 6G, white-dotted line squares, and Figure 6H).

### Expression of IntegriSense probe within tumor tissue

**Figure 7:**
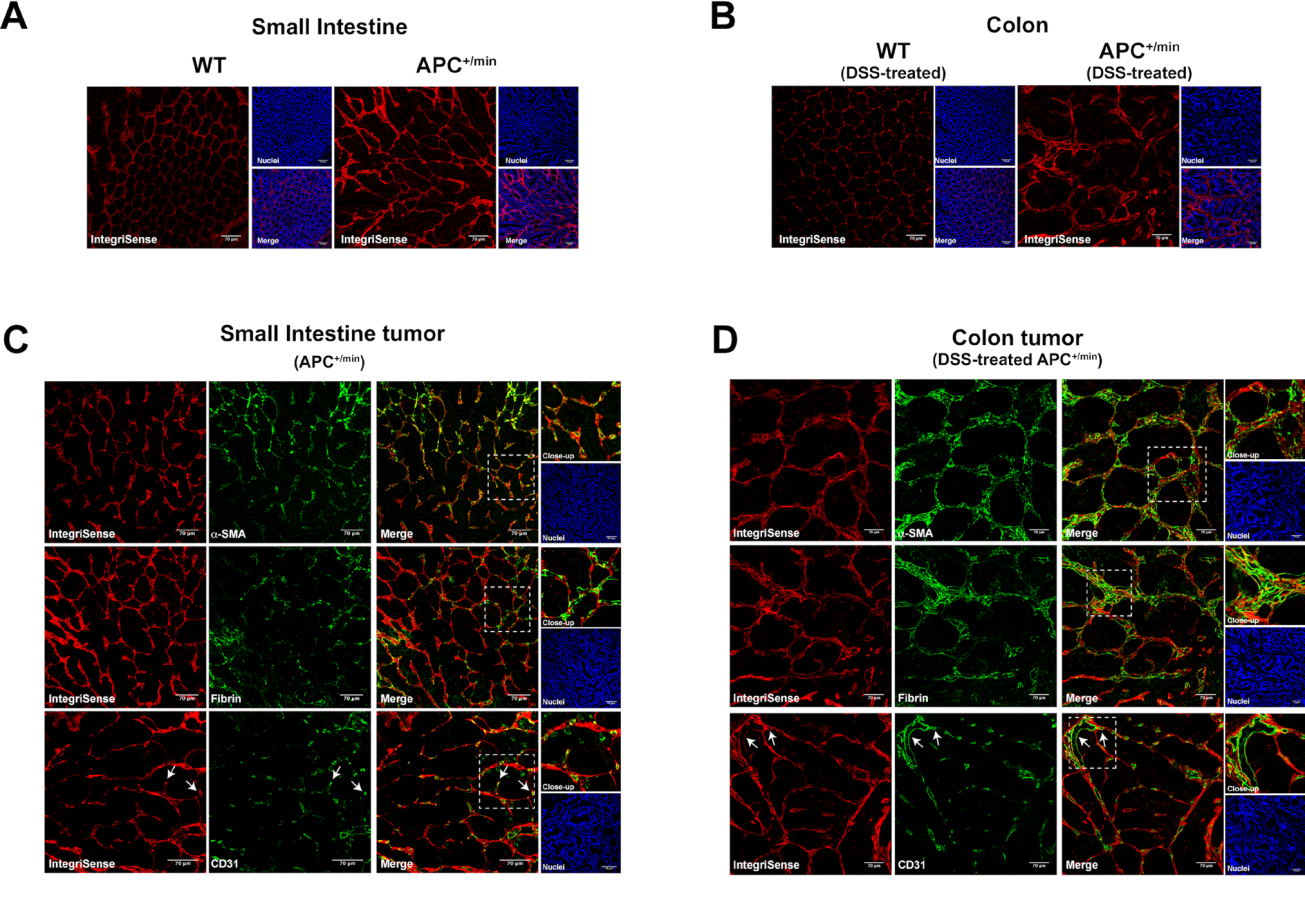
Confocal microscopy analysis of IntegriSense expression in small intestine adenomas and colorectal cancer (**A**) Expression of IntegriSense680 in small intestine tissues of 18 weeks old APC^+/min^ mice and age-related WT mice. Red: IntegriSense680, Blue: DAPI (nuclei). Original magnification 20×. (**B**) Expression of IntegriSense680 in colon tissues of DSS-treated WT and DSS-treated APC^+/min^ mice (as indicated in M&M). Red: IntegriSense680, Blue: DAPI (nuclei). Original magnification 20×. (**C**) Colocalization between IntegriSense680 (red) and α-SMA, Fibrin and CD31 (green) in small intestine tumors of APC^+/min^ mice. Yellow spots indicate colocalization. White-dotted line squares indicate the close-up. White arrows indicate the colocalization between IntegriSense680 (red) and the CD31^+^ tumor-associated vessels (green). Blue: DAPI (nuclei). Original magnification 20×. (**D**) Colocalization between IntegriSense680 (red) and α-SMA, Fibrin and CD31 (green) in colorectal tumors of DSS-treated APC^+/min^ mice. Yellow spots indicate colocalization. White-dotted line squares indicate the close-up. White arrows indicate the colocalization between IntegriSense680 (red) and the CD31^+^ tumor-associated vessels (green). Blue: DAPI (nuclei). Original magnification 20×.

Integrin αvβ3 is probably the integrin most strongly involved in the regulation of angiogenesis. Moreover, it is extensively reported that it binds to a wide range of extracellular matrix (ECM) proteins. To investigate the expression pattern of IntegriSense probe, mice bearing tumors in the small intestine or in the colon were IV injected with 0.08 nmol/g of body weight of IntegriSense680 (Perkin Elmer), a probe analogous to the IntegriSense750 previously used but emitting a fluorescent signal at 680 nm wavelength. After 24 h, mice were sacrificed and small intestine and colon collected. *Ex vivo* IVIS imaging confirmed that the expression pattern of IntegriSense680 was comparable to the one previously observed with IntegriSense750 (Supplementary Figure 1A and 1B). Subsequently, tissues were analysed by immunofluorescence: confocal microscopy analysis confirmed the upregulation of IntegriSense probe in neoplastic tissues (APC^+/min^) compared to healthy mucosa (WT), both in small intestine and colon tumors (Figure 7A and 7B, respectively). Of note, the localization of the probe was similar between small intestine tumor and colon tumors.

To confirm the colocalization of IntegriSense probe with ECM proteins, tissues were analysed for the expression of α-smooth muscle actin (α-SMA) and fibrin: as expected, we observed several spots of colocalization between IntegriSense and both α-SMA and fibrin in small intestine and colon tumors (Figure 7C and 7D, respectively).

As extensively reported in the literature, integrin αvβ3 is strongly involved in the regulation of angiogenesis. We therefore stained tissues with an anti-CD31 antibody to specifically identify vessels: white arrows in Figure 7C and 7D indicate the colocalization between IntegriSense and CD31^+^ vessels, confirming the expression of integrin αvβ3 by tumor-associated vessels in both small intestine and colon tumor (Figure 7C and 7D, respectively).

### *In vivo* monitoring of small intestine and colon cancers

The main advantage of the non-invasive *in vivo* imaging technique is the possibility to monitor deep tumor growth without the need to sacrifice mice. We therefore investigated if the expression of integrin αvβ3 could be used as a marker to follow both small intestine tumor and colon cancer progression.

**Figure 8:**
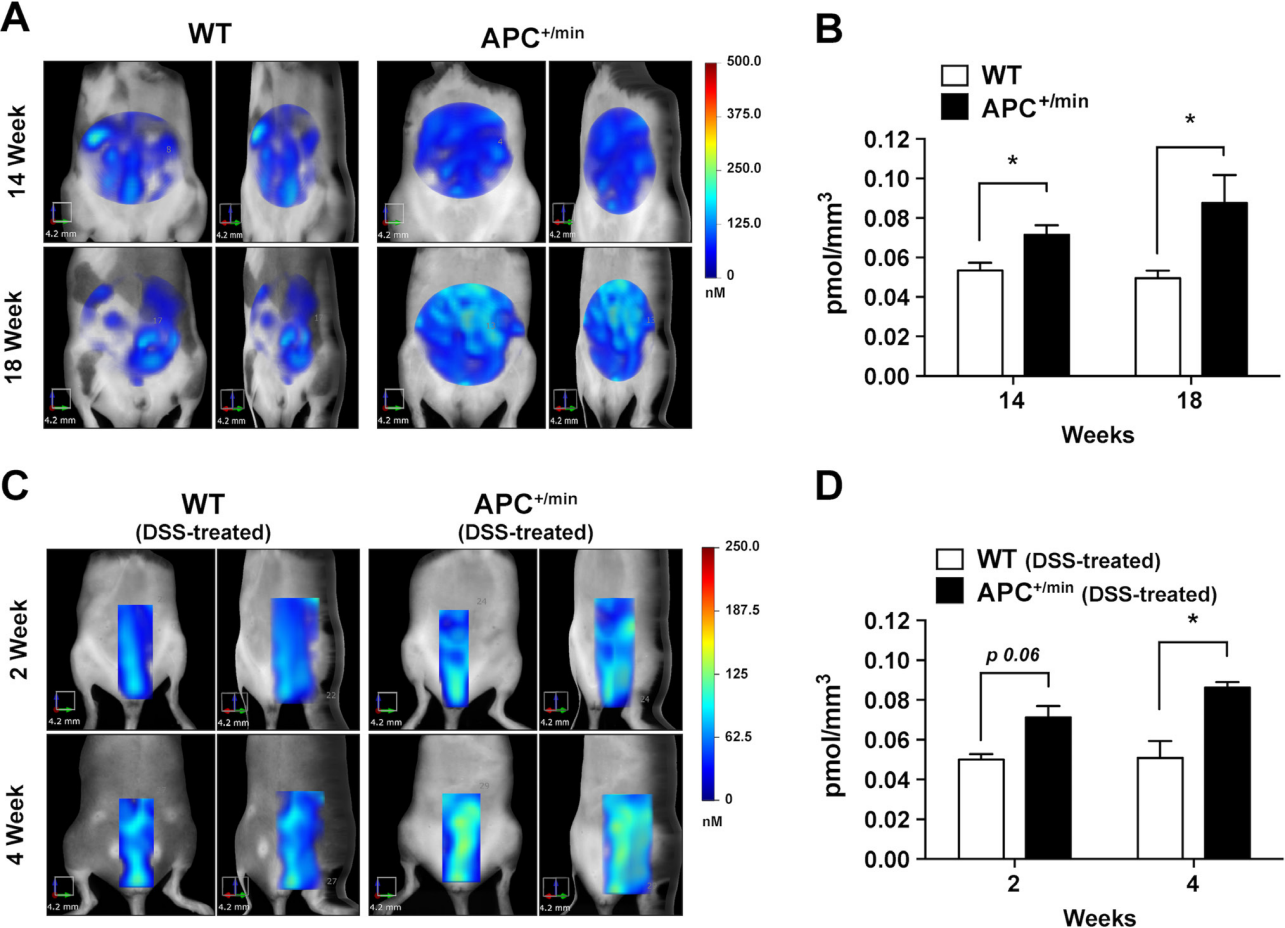
*In vivo* FMT monitoring of small intestine adenomas and colorectal tumor growth (**A**) *In vivo* Fluorescent Molecular Tomography (FMT) analysis of IntegriSense750 accumulation in the small intestine of APC^+/min^ and WT mice. Mice were acquired at 14 and 18 weeks of age. Region of interest (ROI) was tri-dimensionally defined to outline the small intestine. The colorbar shows the concentration (in nM) of the IntegriSense750 probe. (**B**) FMT quantification of IntegriSense750 probe accumulation in the small intestine of APC^+/min^ and WT mice. Mice were acquired at 14 and 18 weeks of age. The total amount of probe within the ROI (in pmoles) was calculated as indicated in the M&M. The amount of pmoles of the probe was normalized based on the volume (mm^3^) of the ROI, after adjustment using the statistically derived threshold value. Data are presented as mean ± SEM. Multiple *t* test, ^*^p < 0.05 (**C**) *In vivo* Fluorescent Molecular Tomography (FMT) analysis of IntegriSense750 accumulation in the colon of the DSS-treated APC^+/min^ and DSS-treated WT mice. Mice were acquired at week 2 and 4 after the end of the DSS administration. Region of interest (ROI) was tri-dimensionally defined to outline the colon. The colorbar shows the concentration (in nM) of the IntegriSense750 probe. (**D**) FMT quantification of IntegriSense750 probe accumulation in the colon of DSS-treated APC^+/min^ and WT mice. Mice were acquired at week 2 and 4 after the end of the DSS administration. The total amount of probe within the ROI (in pmoles) was calculated as indicated in the M&M. The amount of pmoles of the probe was normalized based on the volume (mm^3^) of the ROI, after adjustment using the statistically derived threshold value. Data are presented as mean ± SEM. Multiple *t* test with Holm-Sidak multiple comparison, ^*^p < 0.05.

For the small intestine tumor model, independently from the CX_3_CR1 status, APC^+/min^ and WT mice were shaved and injected, 24 h before the acquisition, with 0.08 nmol/g of body weight of IntegriSense750 probe after 14 and 18 weeks of age. As shown in Figure 8A and 8B, a significant increased accumulation of IntegriSense750 was already detectable in 14 weeks-old APC^+/min^ mice compared to age-matched WT mice; in addition, we observed a close to significant (*p = 0.28*) signal increase from 14 to 18 weeks, a result presumably compatible with a progression of the disease, while no difference was found over time in WT mice (Figure 8A and 8B).

In the colon cancer model, a close to significant higher IntegriSense750 accumulation was already detectable 2 weeks after the end of the DSS administration (Figure 8C and 8D, *p = 0.06)*. Interestingly, IntegriSense750 accumulation increased between the second and the fourth week after DSS administration in APC^+/min^ tumor-bearing mice, while no differences were observed in WT mice. Of note, at this latter time point (4 weeks), IntegriSense750 accumulation is significantly higher in DSS-treated APC^+/min^ tumor-bearing mice compared to DSS-treated WT mice (Figure 8C and 8D). These results indicate that IntegriSense750 probe can be used to monitor tumor growth in both small and large intestine tumor progression.

## DISCUSSION

The identification of new tumor biomarkers increases the opportunity of an early detection and the possibility of a more effective therapeutic intervention [25]. In this study, we demonstrated that integrin αvβ3 is overexpressed in intestinal lesions compared to corresponding healthy mucosa and its visualization by Fluorescent Molecular Tomography (FMT) can be used for the early *in vivo* detection of both small and large intestine tumors. We found that the commercially available probe IntegriSense (Perkin Elmer) [21] specifically accumulates in cancer tissues compared to healthy mucosa, allowing tumor visualization and the monitoring of neoplastic progression in a genetically-induced mouse model of the small intestine tumor (APC^+/min^) as well as in a chemically-induced model of colon cancer (DSS-treated APC^+/min^ mice). In addition, data obtained from *in vivo* FMT detection of IntegriSense correlated with *ex vivo* imaging acquisitions. Moreover, subsequent immunofluorescence analysis by confocal microscopy revealed a high specificity of IntegriSense signal, showing a precise colocalization with ECM proteins and markers of vascularization.

Collectively, these results strongly support the potential utility of the IntegriSense probe and, more in general, of integrin αvβ3 expression in studies aimed at evaluating intestinal tumor occurrence and progression.

The application of optical molecular imaging techniques in preclinical studies is becoming increasingly important, providing the possibility to investigate tumor progression in small animal models in relatively inexpensive, simple and fast way [4, 5, 7]. Colorectal cancer, a major cause of morbidity and mortality in Western countries, has been investigated in the last years using optical imaging approaches [26, 27]. A variety of NIR-conjugated probes have been generated to recognize specific target involved in CRC progression and eventually evaluate the efficacy of therapeutic treatments. However, the majority of these studies have the limitation of having been performed using transplanted orthotopic or xenograft mouse model of CRC [28–34]. In addition, several fluorescent probes have been already used for the detection of chemically-induced mouse models of CRC, but only for *ex vivo* validation of tumor occurrence or metastatic dissemination [35, 36]. More recently, a protease-activatable NIRF imaging probe has been used for the FMT imaging of azoxymethane (AOM)-induced colonic tumors, presenting anyway some limitations as a valid non-endoscopic method for cancer detection [37]. Of note, the advantage of FMT versus conventional 2D optical imaging has been already discussed [9] and recently applied for the detection and quantification of inflammation in a mouse model of inflammatory bowel disease [38].

Although integrin αvβ3 has been already used to visualize transplanted mouse model of CRC [28, 30], to our knowledge this is the first study where its detection by optical *in vivo* imaging has been used for the non-invasive evaluation of genetic or chemically-induced mouse model of intestinal tumor. In addition, the use of fluorescent molecular tomography (FMT) technique, combined with the 750 nm wavelength NIRF probe, allows a more precise three-dimensional and deep localization of the signal and the quantification of the fluorescent probe within the outlined regions of interest. Anyway, the anatomical resolution of optical *in vivo* imaging techniques is quite limited due to light scattering and absorption by surrounding tissues: in the present study, two different ROIs were used in order to isolate as much as possible the small intestine (located in the anterior region of the abdominal cavity and identified with an ellipsoid ROI) and the colon (located in the posterior region of the abdominal cavity, in cranio-caudal direction, and identified with a cylindrical ROI). For this reason, *ex vivo* optical imaging was used as a confirmation of the signal detected by *in vivo* FMT imaging. While a clear correlation between *in vivo* and *ex vivo* imaging was found for the small intestine, a poor but still significant relationship between *in vivo* and *ex vivo* analysis was found for the colon. This difference can be explained by considering the deep position of the colon in the abdominal cavity and the consequent effect of scattering and absorption by surrounding tissues affecting both excitation and emission light, that eventually limits the specificity of the results. These aspects have to be carefully considered, suggesting cautions in the interpretation of *in vivo* imaging results.

We also evaluated the feasibility of IntegriSense probe to distinguish mouse models with a different tumor load. For this purpose, APC^+/min^ mice were previously breed with CX_3_CR1^+/-^ and CX_3_CR1^-/-^ mice. CX_3_CR1 is considered a marker for resident intestinal macrophages, which play a central role in regulating inflammation, and its absence has been correlated with dysregulated inflammatory response, tissue damage and higher tumor occurrence [18–20, 22, 39]. Our data, in line with the literature, showed that in the APC^+/min^ mouse model of small intestine carcinogenesis, the accumulation of IntegriSense probe was slightly but significantly higher in APC^+/min^-CX_3_CR1^-/-^ compared to APC^+/min^-CX_3_CR1^+/-^, confirming the protective effect of CX_3_CR1 in intestinal inflammation.

Furthermore, we compared optical imaging results with conventional histological analysis. Even if a correlation between histology and optical imaging analysis was found (especially in the small intestine carcinogenesis model), this result needs to be carefully discussed. In fact, while optical imaging allows the visualization and analysis of the entire tissue, histology is limited by the physical sectioning of the sample. This sectioning could result in the impossibility to have all tumors in the same slide, especially when bigger tissues and small lesions are considered (as indeed occurs in the APC^+/min^ model of small intestine carcinogenesis). Of note, this consideration could also explain the fact that the slightly difference in small intestine tumor formation between APC^+/min^-CX_3_CR1^-/-^ and APC^+/min^-CX_3_CR1^+/-^ detected by both *in vivo* and *ex vivo* optical imaging was not confirmed by histological analysis (data not shown). In this context, optical imaging and histology can be considered as two complementary, rather than overlapping, techniques, able to provide different features of the same sample.

The role of integrins in cancer progression has been extensively described, being involved in tumor cell migration, invasion and control of angiogenesis, lymphangiogenesis and inflammation [10, 11]. Among integrins, αvβ3 is probably the most strongly involved in the regulation of angiogenesis [11, 13, 40]. It has been reported that, differently from the quiescent endothelium, integrin αvβ3 is highly expressed by tumor-associated vessels, possibly mediating the interaction between endothelial cells and provisional matrix protein, such as vitronectin, fibrinogen and proteolized collagen [11, 15, 41, 42]. These observations are in line with our confocal acquisitions, showing a colocalization between the IntegriSense probe and the matrix protein (α-SMA and fibrin) as well as the CD31^+^ tumor-associated vessels.

The expression of integrin αvβ3 correlated with disease progression in various type of cancer, including breast, pancreatic and prostate cancer and glioblastomas [43–49]. Given its role in cancer progression and neo-angiogenesis processes, it is not surprising that integrin αvβ3 represents an interesting target for the visualization and treatment of a variety of tumors [15, 40, 50–56]. Coupling integrin αvβ3 antagonists or antibodies (LM609) to paramagnetic contrast agents or radionuclides provided the detection of vascularization in experimental tumor models [15, 56–58]. In addition, combination of ^18^F-galacto-RGD and PET allowed the quantification of integrin αvβ3 expression in murine tumor models and in cancer patients [59]. In CRC, it has been demonstrated that vascular expression of integrin αvβ3 correlated with the presence of liver metastasis, reduced relapse-free interval and patients’ overall survival [12]. In addition, treatment with an integrin αvβ3 antagonist resulted in prolonged survival in an orthotopic murine model of colon cancer liver metastasis, with a reduction of vessels in liver metastasis, an increase in endothelial cell apoptosis and a significant decrease in pericyte coverage [60]. Moreover, optical *in vivo* imaging detection of integrin αvβ3 has been successfully used in combination with PET/CT analysis for the visualization of subcutaneous and orthotopic transplanted mouse model of CRC [28]. Accordingly, our *in vivo, ex vivo* and confocal data showed that integrin αvβ3 is already expressed by intestinal tissue, and its expression strongly increased in tumors. These observations confirmed the role of integrin αvβ3 in intestinal carcinogenesis, additionally showing that its expression could be used for the early tumor detection by FMT in preclinical mouse models. In fact, our results demonstrated that a higher IntegriSense accumulation was already detectable after 14 weeks of age in small intestine adenomas-bearing APC^+/min^ mice and 2 weeks after DSS treatment in colorectal cancer-bearing APC^+/min^ mice. Of note, the signal slightly increased in time, indicating that IntegriSense detection can be used to monitor tumor progression both in the small and large intestine. These observations raise the possibility of using integrin αvβ3 imaging for early detection of human CRC. Of course, the application of fluorescent reporter in human is limited to intra-operative imaging-guided surgery, thus the integrin αvβ3 imaging should be approached with MRI or PET techniques.

In conclusion, our results support the use of integrin αvβ3 detection probe for the *in vivo* FMT and *ex vivo* 2D-imaging detection of tumor in preclinical mouse models of intestinal carcinogenesis. In particular, FMT technique, together with the use of NIRF probe, increases the specificity of tumor detection compared to 2D conventional optical imaging methods, ameliorating the anatomical resolution. Combining integrin αvβ3 FMT application with other imaging techniques, such as microCT or MRI, could improve the specificity and sensitivity of intestinal cancer visualization, laying the foundations for the potential use of integrin αvβ3 expression for the early detection of intestinal tumors in humans.

## MATERIALS AND METHODS

### Animal models

Procedures involving animals and their care conformed to institutional guidelines in compliance with national (4D.L. N.116, G.U., suppl. 40, 18-2-1992) and international law and policies (European Economic Community Council Directive 2010/63/EU, OJ L 276/33, 22.09.2010; National Institutes of Health Guide for the Care and Use of Laboratory Animals, U.S. National Research Council, 2011). All efforts were made to minimize the number of animals used and their suffering. All mice were on C57/B6 background. We compared APC^+/min^-CX_3_CR1^+/gfp^ and APC^+/min^-CX_3_CR1^gfp/gfp^ mice (indicated as APC^+/min^, when the CX_3_CR1 status were not considered) with CX_3_CR1^+/gfp^ and CX_3_CR1^gfp/gfp^ (indicated as WT, when the CX_3_CR1 status were not considered). Since GFP expression was not considered in this study, we indicated heterozygous CX_3_CR1^+/gfp^ mice as CX_3_CR1^+/-^ and knock-out CX_3_CR1^gfp/gfp^ mice as CX_3_CR1^-/-^. Mice were maintained in a specific-pathogen free facility, and given *ad libitum* access to food and water. Where indicated, mice were feed with the specific AIN76A, alfalfa-free rodent diet (Mucedola srl).

### RNA extraction and quantitative real-time PCR

RNA was extracted from adenomas or corresponding healthy mucosa independently. Samples were homogenized in TRIzol (Ambion) using Tissue Lyser II (Quiagen). A quantity amounting to 1 µg total RNA was reverse transcribed using the High-Capacity cDNA Archive kit (Applied Biosystems), according to manufacturer’s instruction. cDNA was analyzed by quantitative real-time PCR, performed on CFX96 Touch™ Real-time PCR detection system (BioRad), using Fast SYBR Green (Applied Biosystems). *Actin* gene was used as internal control. The sequences of primers are as follow:

### Integrin β3

Forward: 5’-CCAGGCTCCTATGGAGACAC-3’,

Reverse: 5’-CCCCGGTTGAACTTCTTACA-3’

### Integrin αV

Forward: 5’-GCTCATGCTTTCTATCCCAC-3’

Reverse: 5’- TTCATCGGGTTTCCAAGGTC-3’

### Actin

Forward: 5’-CCCAAGGCCAACCGCGAGAAG-3’

Reverse: 5’-GTCCCGGCCAGCCAGGTCCAG-3’

### Histological analysis

Tissues were harvested, cleaned from the luminal content, longitudinally opened and rolled up transversely (Swiss roll). Tissues were then fixed in 4% paraformaldehyde (PFA) overnight (O/N) at 4° C and paraffin-embedded. To evaluate the histologic architecture of the tissue and the percentage of the tumor area over the total tissue, 2-µm thick sections were cut and stained with hematoxylin and eosin (H&E stain). Slides were scanned with VS120-S5 Virtual Slide System (Olympus) and analyzed with OlyVIA Software (Olympus): subsequently, we calculated the percentage of global tumor area, by summing the dimensions of the multiple neoplastic lesions and dividing them by the dimension of the entire tissue section.

The following formula was used: Percentage of tumor area: (Global dimension of the neoplastic lesions × 100)/Dimension of the entire tissue section

### Fluorescent molecular tomography (FMT)

Fluorescent molecular tomography (FMT) was performed using FMT2000 system (Perkin Elmer). Briefly, animals were fed with AIN76A, alfalfa-free rodent diet (Mucedola srl) for at least 2 weeks before the acquisition in order to reduce autofluorescence background. 24 h before imaging session, mice were shaved to avoid the interference of the fur and IV injected with 0.08 nmol/g of body weight of IntegriSense750 probe (Perkin Elmer) [21]. Prior to the imaging, mice were anesthetized with a mixture of ketamine and xylazine and positioned in a dedicated imaging cassette. The imaging cassette was adjusted to the proper depth to gently restrain the mice. Mice were inserted in supine position into the heated docking system (∼37° C) into the FMT imaging chamber and trans-illuminated with 750 nm near infrared (NIR) laser. The entire acquisition procedure took approximately 6–8 minute per mouse. The collected images were reconstructed and analysed using TrueQuant3.1 software (Perkin Elmer). Three-dimensional regions of interest (ROI) were designed to identify the small intestine and the colon. Threshold was applied equal to the 40% of the mean fluorescence (in nM) of the control mice. The total amount of probe within the ROI (in pmoles) was automatically calculated relative to the internal standard generated with a known concentration of the appropriate NIR dye, following manufacturer’s instruction. The amount of pmoles of the probe was normalized based on the volume (mm^3^) of ROI, after adjustment using the statistically derived threshold value.

### IVIS Lumina III *ex vivo* imaging

*Ex vivo* imaging was performed using IVIS Lumina III system (Perkin Elmer). Briefly, after *in vivo* FMT analysis, mice were sacrificed, the organs collected and acquired using the following parameters: excitation filter 740 nm, emission filter 790 nm, binning 4 or 8, f/Stop 2. Collected images were analysed using Living Image 4.3.1 software (Perkin Elmer): ROIs were designed in order to appropriately select each organs and radiant efficiency calculated.

### Immunofluorescence

For immunofluorescence staining, tissues were harvested and cleaned from the luminal content. Subsequently, tissues were fixed in 4% PFA, O/N at 4° C, dehydrated in 40% Sucrose in PBS^-/-^ (without Calcium and Magnesium) O/N at 4° C and finally embedded in optimum cutting temperature compound (OCT, Diapath). 6-µm thick frozen section were cut with cryostat. For immunofluorescence staining, sections were incubated with primary antibodies in PBS^+/+^ (with Calcium and Magnesium) containing 2% Bovine Serum Albumin (BSA), 5% Normal Goat Serum, 0.1% Triton X-100 for 1h at room temperature. The following antibodies were used: rabbit polyclonal anti-fibrin(ogen) (Dako); mouse monoclonal anti α-SMA-Cy3 (Sigma-Aldrich); Hamster monoclonal anti-PECAM1 (CD31) (Millipore). Sections were then washed 3 times for 5 minutes in PBS^+/+^, 0.2% BSA, 0.05% Tween-20 and incubated with Alexa Fluor (532, 594, 633)-conjugated species-specific, cross-adsorbed, detection antibodies (Molecular Probes) at room temperature for 1h. After 4 washing in PBS^+/+^, 0.2% BSA, 0.05% Tween-20, cell nuclei were counterstained with DAPI (300 nM, Invitrogen). For IntegriSense detection, mice were injected IV with IntegriSense680 (Perkin Elmer) [21]: after 24 h, mice were sacrificed and tissues harvested and processed as previously described. Tissues were finally mounted with FluorPreserve Reagent (Calbiochem) and acquired with a Leica SP8 STED3X confocal microscope, using a 20×/NA 0.75 multi-immersion objective (Leica). Samples were excited with a 405 Diode CW laser and with a tunable pulsed White Light Laser. Emission filter bandwidths were set up in order to avoid any possible spectral overlap between fluorophores. Collected images were then processed with Fiji ImageJ (National Institutes of Health).

### Statistical analysis

Statistical analysis was performed using GraphPad Prism 6 (GraphPad software). Student’s *t* test, multiple student’s *t* test with Holm-Sidak multiple comparison test and linear regression analysis were used, as indicated in the figure legends. A *p* value < 0.05 was considered as statistically significant.

## SUPPLEMENTARY MATERIALS FIGURE



## Abbreviations

FMT: Fluorescent Molecular Tomography
APC: Adenomatous Polyposis Coli
CRC: Colorectal cancer
NIR: Near-InfraRed
ROI: Region of Interest
ECM: Extracellular Matrix
DSS: Dextran Sulfate-Sodium
