# Supplementary material for: Optical in vivo imaging detection of preclinical models of gut tumors through the expression of integrin αVβ3
Source: Oncotarget. 2018 Jul 31;9(59):31380–96. doi: 10.18632/oncotarget.25826 (PMC6101137; doi:10.18632/oncotarget.25826)
Supplement: Supplementary file 1 [file oncotarget-09-31380-s001.pdf]

Optical *in vivo* imaging detection of preclinical models of gut tumors through the expression of integrin αVβ3

SUPPLEMENTARY MATERIALS

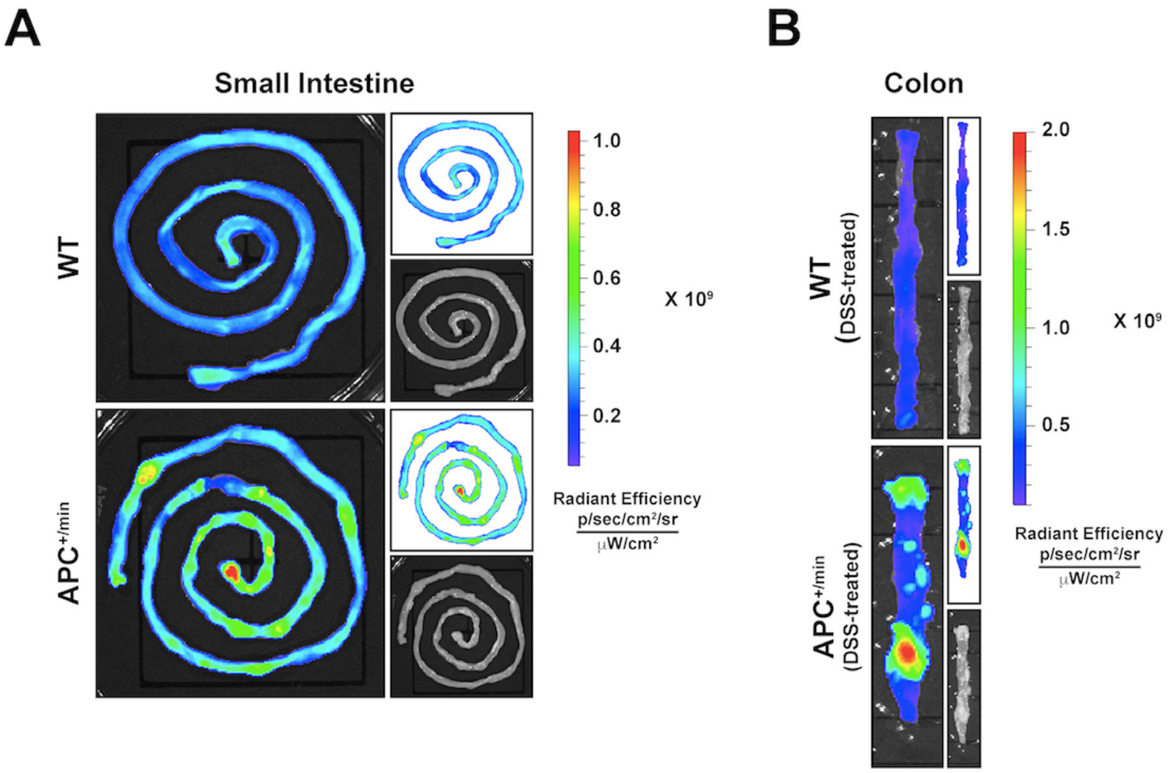

Supplementary Figure 1: *Ex vivo* imaging of IntegriSense680 accumulation in small intestine adenomas and colorectal cancer. (**A**) *Ex vivo* imaging analysis of IntegriSense680 accumulation in the small intestine of 18 weeks old $APC^{+/min}$ and WT mice (**B**) *Ex vivo* imaging analysis of IntegriSense680 accumulation in the colon of DSS-treated $APC^{+/min}$ and WT mice.
